# Metronomic chemotherapy: bridging theory to clinical application in canine and feline oncology

**DOI:** 10.3389/fvets.2024.1397376

**Published:** 2024-06-06

**Authors:** Gonçalo N. Petrucci, Tomás Rodrigues Magalhães, Márcia Dias, Felisbina Luísa Queiroga

**Affiliations:** ^1^Onevet Hospital Veterinário do Porto, Porto, Portugal; ^2^Animal and Veterinary Department, University Institute of Health Sciences, CESPU, CRL, Gandra, Portugal; ^3^Animal and Veterinary Research Centre (CECAV), University of Trás-os-Montes and Alto Douro, Vila Real, Portugal; ^4^Department of Veterinary Sciences, Center for Investigation Vasco da Gama (CIVG), Vasco da Gama University School (EUVG), Coimbra, Portugal; ^5^Department of Veterinary Sciences, University of Trás-os-Montes and Alto Douro, Vila Real, Portugal; ^6^Associate Laboratory for Animal and Veterinary Sciences (AL4AnimalS), University of Trás-os-Montes and Alto Douro, Vila Real, Portugal; ^7^Centre for the Study of Animal Science, CECA-ICETA, University of Porto, Porto, Portugal

**Keywords:** cat, chemotherapy, cytotoxic drugs, dog, metronomic chemotherapy, review

## Abstract

Veterinary oncology has experienced significant evolution over the last few decades, with chemotherapy being currently applied to several neoplasms with therapeutic success. Traditionally, chemotherapy protocols are based on classic cytostatic drugs under the concept of maximum tolerated dose (MTD), which has been associated with a greater risk of toxicity and resistance. Thus, new therapeutic alternatives have emerged, such as metronomic chemotherapy (MC), introducing a new paradigm in cancer treatment. MC consists of administering low doses of chemotherapy drugs continuously over a long period of time, modulating the tumour microenvironment (TME) due to the combination of cytotoxic, antiangiogenic and immunomodulatory effects. This multi-targeted therapy has been described as a treatment option in several canine and feline cancers since 2007, with positive results already published in the literature, particularly in mammary carcinomas and soft tissue sarcomas in dogs. The aim of this review article is to describe the current knowledge about the use of MC in small animal oncology, with emphasis on its mechanisms of action, the most commonly used drugs and clinical outcome.

## 1 Introduction

The prevalence of neoplastic disease in companion animals has been increasing over the past few years. This phenomenon may be attributed, in part, to the enhanced longevity observed in pets, which makes them more susceptible to developing age-related diseases, like cancer (1, 2). Thus, effective treatment strategies are of paramount importance in veterinary oncology.

Anticancer drugs have been administered according to the “maximum-tolerated dose” (MTD) concept, whose limit is related to the toxic effects on the patient’s healthy tissues (3–5). In an attempt to overcome some of these limitations, there was a need for new therapeutic strategies that would allow tumour control with fewer adverse effects, which led to the development of metronomic chemotherapy (MC). This new chemotherapeutic modality emerged as a result of several research studies that showed that some anticancer drugs had superior efficacy when used continuously in lower doses than as part of conventional chemotherapy regimens (6, 7). This advantage was later realised to result from the antiangiogenic action of these cytostatics when administered under this regimen, showing a new and promising therapeutic target beyond direct cytotoxicity (8).

Although existing literature does not offer conclusive evidence to establish definitive therapeutic protocols, this review aims to present a comprehensive overview of the current state of MC in small animal practice. By synthesising available data and major findings, this review seeks to clarify MC’s current utilisation and potential benefits, contributing insights towards the advancement of veterinary oncological care.

## 2 Metronomic chemotherapy: from definition to action-driven effects

The term “metronomic chemotherapy” was proposed by Douglas Hanahan in the early 2000s (9), however the first steps towards the development of this novel therapy began three decades earlier when Judah Folkman suggested a potential therapeutic effect of inhibiting tumour neovascularisation (10, 11). Following this hypothesis, Baguley et al. (12) proved a few years later that chemotherapeutic agents were able to reduce the blood flow of drug-resistant tumours in mice, which in turn motivated the experimental evaluation of different schedules. Two of the most important studies of that time were conducted by Browder et al. (6) and Klement et al. (7), who showed, respectively, that protocols with a higher frequency of administration and that used low-dose drugs continuously, increased the deleterious effect on tumour endothelial cells, inducing apoptosis and, consequently, tumour regression. Further advantages were subsequently identified, particularly lower toxicity compared to conventional chemotherapy and greater efficacy in overcoming chemoresistance (6, 13). Driven by these promising discoveries and an increasingly in-depth knowledge about cancer and the tumour microenvironment (TME), this concept has evolved from preclinical studies to clinical trials on human and animal patients (14, 15).

**Figure 1:**
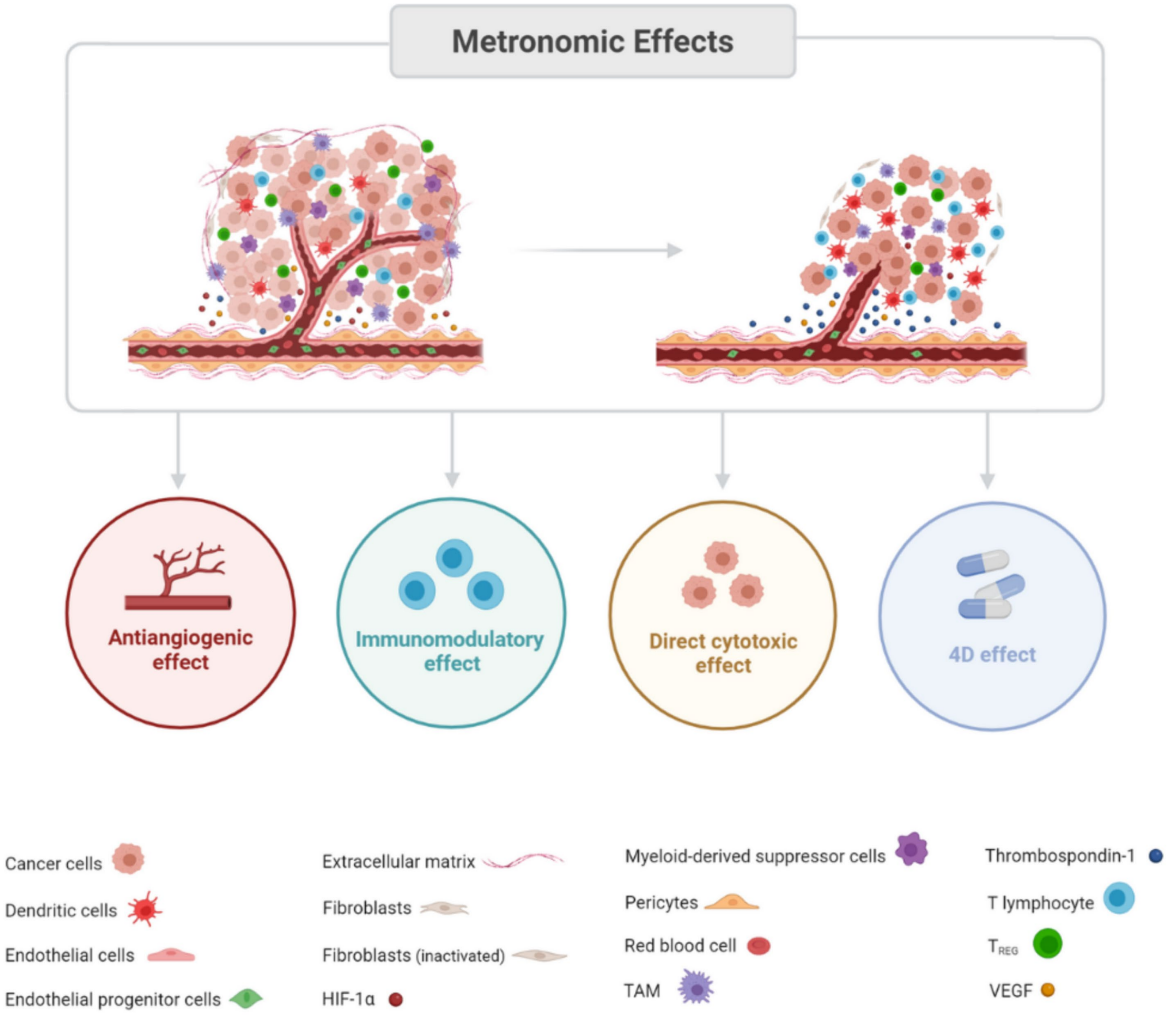
Schematic illustration of the effects of metronomic chemotherapy on a tumour. Metronomic chemotherapy influences tumour growth through multiple mechanisms. Antiangiogenic effect: this includes inhibition of endothelial and endothelial progenitor cell proliferation and circulation, reduction in the differentiation of immature endothelial cells, downregulation of proangiogenic factors like VEGF and HIF-1α, and upregulation of antiangiogenic factors such as thrombospondin-1. Immunomodulatory effect: this effect is characterised by the suppression of regulatory T (Treg) lymphocytes and myeloid-derived suppressor cells (MDSCs), alongside the promotion of dendritic cell activation, cytotoxic T cells, and natural killer (NK) lymphocytes. Direct cytotoxic effect: metronomic chemotherapy directly targets tumour cells, reducing cancer stem cell populations and inducing a state of tumour dormancy. 4D effect: this involves modulating tumour resistance and triggering cancer cell death through drug-driven dependence and deprivation mechanisms.

Nowadays, MC can be defined as the continuous administration of cytostatic agents at low and minimally toxic doses without prolonged rest periods and, despite initially being conceived. Despite initially being conceived as an antiangiogenic therapy, its scope has since expanded, including a multi-targeted strategy that impacts not only the tumour endothelium but also exerts immunomodulatory effects, directly inhibits tumour proliferation, and induces a state of neoplastic dormancy (Figure 1) (14, 16).

### 2.1 Antiangiogenic effect

Tumour neovascularisation, a pivotal process for tumour growth and metastasis, involves angiogenesis and vasculogenesis (17). By definition, angiogenesis corresponds to new vascularisation generated from mature endothelial cells in existing vessels, whereas in vasculogenesis blood vessels arise from endothelial progenitor cells (EPCs) of the bone marrow (18–20). In fact, tumours may employ both mechanisms simultaneously (21). To foster endothelial proliferation and new vessel formation, tumours trigger the release of proangiogenic substances and suppress endogenous antiangiogenic factors like endostatin and thrombospondin-1 (TSP-1) (2, 19, 22, 23). This phenomenon, called “angiogenic switch,” enables tumours to exit their dormant state, occurring at diverse tumour progression stages (17, 19, 24). Hypoxia is one of its main triggers, since low concentrations of oxygen in the TME typically promote the production of proangiogenic factors (22). Several of these factors have already been identified, including vascular endothelial growth factor (VEGF), basic fibroblast growth factor (bFGF), platelet-derived growth factor (PDGF), transforming growth factor-β (TGF-β), hypoxia-inducible factor-1α (HIF-1α) and angiopoietin-1 (25–29).

These antiangiogenic effects are evidenced by studies suggesting that tumour angiogenesis and vasculogenesis can be inhibited by MC through multiple pathways, including the reduction of endothelial and EPC proliferation and circulation, hindering immature endothelial cell differentiation, and modulating proangiogenic and antiangiogenic factors (6, 15, 30–33).

The administration of immunostimulating cytokines, such as interleukin (IL)-12 can potentially enhance the antiangiogenic properties of MC, according to experimental studies in mice (34, 35). Evidence of this benefit in companion animals is still scarce, but a preliminary study showed promising therapeutic results in a small group of dogs (23).

Finally, considering that some VEGF isoforms have been associated with the formation of new intra- and peritumoral lymphatic vessels, it could be hypothesised that MC may also have a crucial effect on inhibiting lymphangiogenesis, preventing neoplastic spread through the lymphatic route (36, 37).

### 2.2 Immunomodulatory effect

Neoplasms have several strategies to escape the immune surveillance, such as the activation of myeloid-derived suppressor cells (MDSCs) and regulatory T-cells (Tregs) that induce an immunosuppressive state in the TME (19, 22). In turn, these cells contribute to immune evasion and tumour progression by promoting macrophage and neutrophil polarisation, compromising the activation of dendritic cells, suppressing effector cells (e.g., cytotoxic and helper T-cells and natural killer cells) and stimulating the secretion of immunosuppressive cytokines such as interleukin (IL)-10 and transforming growth factor-β (TGF-β) (22, 27, 32, 38).

According to several studies, MC has as an immunomodulatory effect, counteracting the aforementioned immune evasion strategies, namely suppressing MDSCs and Tregs function, increasing lymphocyte, memory T-cell and natural killer cell proliferation and upregulating dendritic cells (23, 39–44).

### 2.3 Direct cytotoxic effect

Another mechanism of action that has been attributed to MC is the direct cytotoxic effect on tumour cells, particularly cancer stem cells (CSCs) (14, 15, 45, 46). These CSCs, known for their role in therapeutic resistance through their capacity for self-renewal and differentiation into diverse cancer cell types, are fundamental in tumour proliferation, invasion, and metastasis (47). Unlike traditional high-dose chemotherapy, MC has demonstrated efficacy in diminishing CSC populations, potentially by limiting angiogenesis and directly influencing VEGF expression (48, 49).

Additionally, MC’s impact extends to the three compartments of the tumour microenvironment (immune system, tumour cells and vasculature). The intricate interplay within these compartments may induce and maintain a state of tumour dormancy, a dynamic stability between cell proliferation and cellular apoptosis, potentially ensuring long-term asymptomatic control of the disease (15, 32, 50, 51).

### 2.4 The 4D effect

The drug-driven dependence/deprivation effect (also known as the 4D effect) can be achieved through long-term exposure of the tumour to cytotoxic agents followed by abrupt withdrawal (52). This effect, as per *in vitro* studies, leaves drug-dependent cells more hypersensitive and thus more vulnerable to therapeutic strategies, a phenomenon observed in breast cancer cells resistant to anti-hormonal treatments (53, 54). Hence, it is suggested that a temporary interruption introduced after a prolonged course of chemotherapy, a strategy frequently employed in MC protocols, could break tumour resistance and trigger cancer cells death (52–54). This approach suggests a strategic manipulation of drug administration to enhance treatment efficacy, optimising cancer therapy. Nonetheless, the 4D effect has only been investigated in human medicine, so further research is required to validate the advantages of this effect in *in vivo* animal models undergoing MC protocols.

## 3 Metronomic chemotherapy in veterinary oncology

The use of MC in small animal practice was described for the first time in 2007 in a group of dogs diagnosed with splenic hemangiosarcoma, which were treated with a continuous low-dose oral chemotherapy protocol that included cyclophosphamide, etoposide and piroxicam (55). Low-dose metronomic cyclophosphamide was also later described in feline patients diagnosed with different spontaneous malignancies, such as sarcomas and carcinomas (56). Since then, this modality has been increasingly applied to veterinary patients due to the fewer side effects, less need for supportive medications, generally low cost, less stressful administration, convenience to pet owners, and possible combination with other therapies (14). Although initially it was arguably considered by some authors as a merely palliative treatment, it is currently known that MC has greater therapeutic potential, whether in combination with surgery (57–66), radiotherapy (67, 68) or electrochemotherapy (66), or even as first-line treatment for advanced, metastatic or incurable disease (69, 70). Furthermore, its use in combination with MTD chemotherapy (MTDC) has also been reported, either simultaneously or after the latter for maintenance therapy (chemo-switch regimen) (57, 60, 62, 71–76).

### 3.1 Drugs, doses and schedules

Several cytotoxic drugs have been used in MC protocols in veterinary oncology patients over the last few years. The most commonly used is oral cyclophosphamide, whose dose ranges between 6 and 27 mg/m^2^ once daily to once every other day, according to several published clinical trials (23, 42–44, 55–57, 59–67, 69–72, 74–82). Although less frequently, oral chlorambucil has been described as the main drug at the dose of 4 mg/m^2^ daily in dogs (58, 62, 83–85) and 0.4 to 0.6 mg/kg or 4 mg/m^2^ every other day in cats (79, 82) for the treatment of some neoplasms in these two species. It has also been used as a substitute for cyclophosphamide when sterile haemorrhagic cystitis occurs (57, 70, 73, 74, 76, 80). In turn, metronomic prescription of lomustine (68, 86), temozolomide (42), and etoposide (55, 57, 87) has also been described in the oncological treatment of some canine patients at daily doses of 2.84 mg/m^2^, 6.6 mg/m^2^ and 50 mg/m^2^, respectively.

Despite the drug doses and schedules mentioned above, there are currently no standard recommendations for drug doses, as published data are still scarce and quite heterogenous for most canine and feline tumour types. Even so, several authors have shown that the prescribed dose has a significant impact on the patient’s therapeutic response. For example, Burton et al. (43) reported significantly greater immunomodulatory and antiangiogenic effects when using a higher dose of oral cyclophosphamide (15 mg/m^2^ versus 12.5 mg/m^2^) in dogs with soft tissue sarcoma. However, it should be noted that higher doses are often associated with earlier and more frequent manifestation of adverse effects, as has already been described in canine patients treated with higher doses of chlorambucil (6 to 8 mg/m^2^ versus 4 mg/m^2^) (88). Further research is still required to establish the minimum effective drug dose for treating each specific tumour type.

Several clinical trials based on MC have been published in the last two decades, presenting different doses and schedules, as represented in Table 1.

**Table 1:** Metronomic chemotherapy protocols prescribed to veterinary patients in 36 clinical trials.

Reference/study design	Tumour type/N° of animals treated with MC	Main metronomic drug, dose, schedule and duration	Concurrent drugs	Outcome/clinical relevance
Lana et al. (55) Prospective	Splenic hemangiosarcoma 9 dogs	CYC (12.5 to 25 mg/m^2^/day PO), in 3-weeks cycles, alternating with etoposide, for 6 months.	Piroxicam (0.3 mg/kg/day PO) and etoposide (50 mg/m^2^/day PO in 3-week cycles).	Median OST was significantly longer compared to canine patients treated with DOX (178 days versus 133 days, respectively; *p* = 0.03).
Elmslie et al. (77) Retrospective	Soft tissue sarcoma 30 dogs	CYC (10 mg/m^2^/day or EOD PO), on a long-term.	Piroxicam (0.3 mg/kg/day PO).	DFI in dogs treated with adjuvant MC was significantly higher than others (*p* < 0.0001).
Tripp et al. (86) Prospective	Various tumour types 81 dogs	Lomustine (2.84 mg/m^2^/day PO), for a median duration time of 98 days.	NSAID (*n* = 29) or prednisone (*n* = 7).	PR and SD in 6.3 and 29.7% (out of 64 dogs), respectively. Median duration time of SD = 137 days.
Burton et al. (43) Prospective	Soft tissue sarcoma 11 dogs	CYC (12.5 or 15 mg/m^2^/day PO), for 28 days.	None.	Significant decrease in n° and % of Tregs and in tumour MVD, at a dose of 15 mg/m^2^/day.
Marchetti et al. (78) Prospective	Various tumour types 15 dogs	CYC (25 mg/m^2^/day PO), until disease recurrence and progression.	Celecoxib (2 mg/kg/day PO).	CR and SD in 6.7 and 33.3%, respectively. Median OST = 3.39 months. Improved QoL in all animals.
Leach et al. (83) Prospective	Various tumour types 36 dogs	Chlorambucil (4 mg/m^2^/day PO), on a long-term.	COX inhibitors (if previously introduced; *n* = 12).	CR, PR and SD in 8.3, 2.8 and 47.2% of dogs, respectively. Median PFI = 61 days and ST = 153 days.
Mitchell et al. (44) Prospective	Various tumour types 13 dogs	CYC (15 mg/m^2^/day PO), for 4 to 6 weeks.	Toceranib (2.75 mg/kg EOD PO) and/or NSAID/pain control drugs.	Significant increase in serum concentration of interferon-gamma. SD in 46.2% and PD in 53.8%.
Schrempp et al. (84) Prospective	Urinary bladder TCC 31 dogs	Chlorambucil (4 mg/m^2^/day PO), on a long-term.	COX inhibitors (if previously introduced; *n* = 25).	Median PFI = 119 days and median ST = 221 days. PR in 3.3% and SD in 66.7% (out of 30 dogs).
Bracha et al. (71) Retrospective	Appendicular osteosarcoma 30 dogs	CYC (10 to 12 mg/m^2^/day PO), on a long-term.	CM_group_: piroxicam + carboplatin (300 mg/m^2^ IV q3 weeks); ACM_group_: piroxicam, carboplatin and DOX (30 mg/m^2^ q3week IV).	No significant difference in DFI (*p* = 0.811) or ST (*p* = 0.918) between groups. Median ST = 217 days and 189 days for the CM group (*n* = 14) and the ACM group (*n* = 16), respectively.
Leo et al. (56) Retrospective	Various tumour types 24 cats	CYC (6 to 27 mg/m^2^/day, EOD or twice a week PO), for at least 1 month.	NSAID (*n* = 18), toceranib (2.5 mg/kg three times a week; *n* = 4) +/− thalidomide (5 mg/cat/day; n = 6).	Median PFS was 90 days and 297 days, depending on whether MC was used as palliative or adjuvant treatment, respectively.
Spugnini et al. (79) Prospective	Various tumour types 22 dogs and 2 cats	Dogs: CYC (12.5 mg/m^2^/day PO). Cats: Chlorambucil (4 mg/m^2^ EOD PO); until CR or absence of disease for 1 year.	Piroxicam (0.3 mg/kg/day or EOD PO) + lansoprazole (1 or 5 mg/kg/day PO) + water alkaliser.	PR or CR in 75% (18 out of 24). High dose lansoprazole associated with a water alkaliser increased the therapeutic response to MC.
London et al. (80) Prospective	Appendicular osteosarcoma 81 dogs	CYC (10 mg/m^2^ EOD PO), for 8 months or until signs of PD were detected.	Test_group_: toceranib (2.75 mg/kg EOD PO) + piroxicam (0.3 mg/kg EOD PO); Control_group_: piroxicam.	No significant difference in DFI (*p* = 0.274) or OST (*p* = 0.08). Median OST = 318 days (test_group_; *n* = 46) and 242 days (control_group_; *n* = 35).
Wendelburg et al. (57) Retrospective	Splenic hemangiosarcoma 26 dogs	MC_group_ (*n* = 13): CYC (9.3 to 16 mg/m^2^/day PO); MC + MTDC_group_ (*n* = 13): CYC (9.2 to 12 mg/m^2^/day PO), on a long-term/until death.	MC_group_: NSAID (*n* = 12) and/or etoposide (50 mg/m^2^/day PO; *n* = 1); MC + MTDC_group_: DOX (25 or 30 mg/m^2^ q2-3 weeks IV) + NSAID.	MTDC and/or MC appear to prolong survival compared with surgery alone, but only in the first 4 months (*p* = 0.018). MC and MTD appear to be more effective combined than each alone.
Cancedda et al. (67) Retrospective	Soft tissue sarcoma 20 dogs	CYC (7 mg/m^2^ EOD PO), on a long-term.	Thalidomide (1 to 2 mg/kg/day PO) and piroxicam (0.3 mg/kg/day PO).	MC + radiation allowed a significantly longer median ST (757 days) than radiation alone (286 days).
Rasmussen et al. (72) Prospective	Various tumour types 13 dogs (phase I) and 8 dogs (phase II)	CYC (10 to 15 mg/m^2^/day PO), for a median duration time of 84 days in phase 1 and 42 days in phase 2.	DOX (30 mg/m^2^ IV q3weeks) for a median of 4 or 2 treatments (phase I or II, respectively).	MC plus DOX led to depletion of circulating lymphocytes and absolute Tregs, but with no significantly superior effect compared to DOX alone.
Finotello et al. (73) Retrospective	Hemangiosarcoma 10 dogs	CYC (7 to 15 mg/m^2^/day or EOD PO), on a long-term.	NSAID (*n* = 10) and thalidomide (2 to 3 mg/kg/day or EOD PO; *n* = 7).	MC + MTDC was significantly associated with longer median TTM (*p* = 0.028) and ST (*p* = 0.030) than MTDC only.
Denies et al. (42) Prospective	Various types of tumours 30 dogs	CYC (12.5 mg/m^2^/day PO), temozolomide (6.6 mg/m^2^/day PO) or both, until death.	NSAID, when needed.	Significant decrease in the % of circulating Tregs with CYC alone (*p* = 0.02) or CYC plus temozolomide (*p* = 0.03), but not with temozolomide alone (*p* = 0.3).
Cicchelero et al. (23) Prospective	Various tumour types 6 dogs	CYC (12.5 mg/m^2^/day PO), until day 35 (at least).	Intratumoral interleukin (IL)-12 electrogene therapy (EGT).	MC + IL-12 EGT was associated with a significant reduction in Tregs (*p* = 0.046), a significant delay in disease progression (*n* = 3) and improved QoL (*n* = 4).
Matsuyama et al. (75) Retrospective	Various tumour types 50 dogs	CYC (25 mg/m^2^ EOD PO), for a median duration time of 90 days.	NSAID (*n* = 45), molecular targeted drugs (*n* = 9), MTDC (*n* = 5), radiation (*n* = 2) and immunotherapy (*n* = 1).	A higher cumulative dose of metronomic CYC was significantly associated with an increased risk of developing SHC (*p* = 0.048).
Matsuyama et al. (74) Retrospective	Splenic hemangiosarcoma 18 dogs	CYC (10, 15 or 25 mg/m^2^/day or EOD PO), for a median duration time of 46 days.	NSAID (*n* = 13). DOX (30 mg/m^2^ or 1 mg/kg IV, given concurrently in 4 dogs and before MC in the remaining ones).	MC after DOX did not significantly improve PFI and OST compared to DOX alone (*p* = 0.563 and *p* = 0.148, respectively).
Duffy et al. (68) Retrospective	Appendicular osteosarcoma 43 dogs	Lomustine (2.84 mg/m^2^/day PO), until death or dose-limiting AEs.	NSAID (*n* = 40).	Adjuvant lomustine was not associated with a significantly longer median ST compared to radiation therapy alone (184 days versus 154 days; *p* = 0.84).
Matsuyama et al. (76) Retrospective	Appendicular osteosarcoma 19 dogs	CYC (15 mg/m^2^/day PO), until tumour progression or dose-limiting AEs (median duration time = 94 days).	NSAID: meloxicam (0.1 mg/kg/day PO; *n* = 18) or firocoxib (6 mg/kg/day PO; *n* = 1).	MC after adjuvant carboplatin was not associated with a significantly longer PFI and OST compared to carboplatin alone (*p* = 0.14 and *p* = 0.24, respectively).
Bentley et al. (58) Prospective	Cerebral glioma 8 dogs	Chlorambucil (4 mg/m^2^/day PO), at least 3 days before surgery and then until death or dose-limiting AEs (median duration time = 258 days).	Prednisone (0.2 to 1 mg/kg/day) and lomustine (60 mg/m^2^/month for 5 months).	MC was considered well tolerated in dogs diagnosed with glioma. Median PFI = 253 days and median OST = 257 days.
Polton et al. (69) Prospective	Primary lung carcinoma 25 dogs	CYC (10 mg/m^2^/day EOD PO), on a long-term.	Piroxicam (0.3 mg/kg/day PO) and thalidomide (2 mg/kg/day PO).	PR in 16% and SD in 76% of dogs. Median TTP (172 days) and ST (139 days) were significantly longer compared to other therapeutic options.
De Campos et al. (59) Prospective	Malignant mammary gland neoplasm 9 dogs	CYC (15 mg/m^2^/day PO), for 6 months or indefinitely when distant metastases were detected.	Firocoxib (5 mg/kg/day PO).	Median ST with MC (431 days) was lower compared to surgery, carboplatin and thalidomide (845 days), but higher compared to surgery only (245 days) or surgery plus carboplatin (242 days).
Alexander et al. (60) Retrospective	Splenic hemangiosarcoma 22 dogs	CYC (12.5 mg/m^2^/day or 25 mg/m^2^ EOD), on a long-term.	NSAID (*n* = 13) +/− doxycycline (*n* = 5).	MC was not associated with a significant improvement in outcome. Median PFS = 185 days and median OST =212 days.
Marconato et al. (61) Retrospective	Splenic hemangiosarcoma 38 dogs	CYC (10 to 15 mg/m^2^/day or EOD PO), for a median duration time of 35 days.	NSAID (*n* = 38) +/− thalidomide (2 to 4 mg/kg/day PO; *n* = 35)	MC was associated with a median TTP (*p* = 0.025) and ST (*p* = 0.023) significantly lower than MTDC.
Treggiari et al. (62) Retrospective	Splenic hemangiosarcoma 43 dogs	MC_group_: CYC (10 to 15 mg/m^2^/day or EOD PO; *n* = 18) or chlorambucil (4 mg/m^2^/day PO; *n* = 2). AMC_group_: CYC, but after adjuvant MTDC.	MC_group_ (*n* = 20): NSAIDs (*n* = 10) and/or thalidomide (2 to 8.7 mg/kg/day PO; *n* = 3). AMC_group_ (*n* = 23): NSAID (*n* = 13).	Median TTP = 222 days and median ST = 225 days in the MC group, which did not differ significantly from the outcome of dogs treated with MTDC or the latter followed by MC (AMC group).
Marconato et al. (81) Prospective	Hepatocellular carcinoma 6 dogs	CYC (10 mg/m^2^/day PO), during a median duration time of 21 days.	Piroxicam (0.3 mg/kg/day PO) and thalidomide (2 mg/kg/day PO).	SD in 50% and PD in 50%. Poor outcome: median TTP = 27 days and median OST = 32 days.
Petrucci et al. (63) Retrospective	Mammary carcinoma 23 cats	CYC (15 mg/m^2^/day PO), for 6 months.	Meloxicam (0.05 mg/kg/day PO).	Median DFI (372 days) and median OST (430 days) with MC were not significantly different compared to surgery alone or surgery plus DOX.
Petrucci et al. (82) Retrospective	Metastatic mammary carcinoma 15 cats	CYC (15 mg/m^2^/day PO; *n* = 11) or chlorambucil (0.4–0.6 mg/kg EOD PO; *n* = 4), for a median duration of 60 days.	Supportive analgesic therapy (meloxicam, buprenorphine and/or gabapentin), when appropriate.	Median TSS = 75 days. No statistically difference was identified in TSS with MC compared to MTDC or toceranib phosphate administration (*p* = 0.197).
Milevoj et al. (70) Retrospective	Malignant oral tumours 12 dogs	CYC (15 to 25 mg/m^2^/day PO), for a median duration time of 156 days.	NSAID (*n* = 6).	PR or SD in 50% after 1 month and in 33.3% after 3 months. Median ST = 155 days.
Gedon et al. (85) Retrospective	Urothelial carcinomas 9 dogs	Chlorambucil (4 mg/m^2^/day PO), for a median duration time of 297 days.	Meloxicam (0.1 mg/kg/day PO)	MST = 445 days, which is significantly longer than oral meloxicam alone (151 days), but significantly shorter compared to surgery (748 days).
Machado et al. (64) Prospective	Mammary carcinoma 8 dogs	CYC (12.5 mg/m^2^/day PO), for 8 months.	Carboplatin (300 mg/m^2^ q3weeks IV), for 6 sessions.	MC plus carboplatin was associated with a significantly increased survival rate compared to carboplatin alone (*p* = 0.0044).
Alonso-Miguel et al. (65) Retrospective	Inflammatory mammary carcinoma 8 dogs	CYC (12.5 mg/m^2^/day PO), until death or dose-limiting AEs.	NSAID and toceranib phosphate (2.4 to 2.7 mg/kg 3x/week PO).	Clinical benefit in 100%. Median OST (*p* = 0.046) and median TTP (*p* = 0.010) were significantly longer with MC compared to COX-2 inhibitor therapy alone.
Garcia et al. (66) Prospective	Oral melanoma 9 dogs	CYC (15 mg/m^2^/day or EOD PO), for 6 months.	Tumour lysate vaccine	Vaccine plus MC was not associated with significantly longer PFS (*p* = 0.294) and OST (*p* = 0.553) compared to vaccine alone.

AEs (adverse effects); COX (cyclooxygenase); CR (complete remission/response); CYC (cyclophosphamide); DFI (disease-free interval); DOX (doxorubicin); EOD (every other day); IL-12 EGT (intratumoral interleukin-12 electrogene therapy); IV (intravenous); MC (metronomic chemotherapy); MTDC (maximum-tolerated dose chemotherapy); MVD (microvessel density); n° (number); NSAID (non-steroidal anti-inflammatory drug); OST (overall survival time); *p* (significance); PD (progressive disease); PFI (progression-free interval); PFS (progression-free survival); PO (per os/mouth); PR (partial remission/response); q (every); QoL (quality of life); SCC (squamous cell carcinoma); SD (stable disease); SHC (sterile haemorrhagic cystitis); ST (survival time); Tregs (regulatory T cells); TTC (transitional cell carcinoma); TTM (time to metastasis); TTP (time to progression); TSS (tumour-specific survival). Symbols: % (percentage).

### 3.2 Combination with other therapeutic drugs

In order to potentiate its antiangiogenic and immunomodulatory effects, MC is often combined with the administration of cytotoxic and non-cytotoxic agents (4, 19, 89).

In the first scenario, MC can be used in combination with MTDC to reduce the risk of neoplastic regrowth between administrations. In fact, several authors have suggested the possibility of an additive and synergistic effect of this combined use, which could potentially improve the prognosis of these patients (57, 72). The two intravenous drugs that have been most commonly used in a MTDC setting, in combination with MC, are doxorubicin (57, 71, 72, 74) and carboplatin (64, 71). Additionally, tyrosine kinase inhibitors have also been prescribed in association with metronomic cyclophosphamide, particularly oral toceranib at a dose of 2.4 to 2.75 mg/kg every other day or three times a week (44, 56, 65, 80, 90).

Regarding non-cytotoxic agents, non-steroidal anti-inflammatory drugs (NSAIDs) are the most prescribed in association with MC, due to their ability to inhibit cyclooxygenase isoform-2 (COX-2), whose expression is considered a negative prognostic factor in various types of canine and feline tumours (91). This inhibitory effect compromises endothelial cell tube formation and VEGF expression, preventing tumour progression (73, 78, 80) Thus, several COX-2 inhibitors have been included in MC protocols, such as piroxicam (55, 56, 61, 62, 67, 69–71, 73–75, 77, 79–81, 86, 90), meloxicam (56, 61–63, 70, 73–76, 82, 85, 86, 90), firocoxib (56, 59, 62, 65, 73, 76, 86, 90), carprofen (68, 70, 86), deracoxib (74, 75, 86), celecoxib (78), and cimicoxib (65). Amongst these, piroxicam, an oxicam derivate, is the NSAID whose efficacy as anticancer drug has been most recognised, at a recommended dose of 0.3 mg/kg per day or every other day (69, 79, 92). There are also a few reported cases of the combined use of MC with corticosteroids, such as prednisone (58, 86).

Other non-cytotoxic drugs that have also been described in patients undergoing MC are thalidomide (56, 61, 62, 67, 69, 73, 81) and doxycycline (60, 74, 75, 90). Thalidomide has been associated with anti-inflammatory and antiangiogenic effects by inhibiting the expression of VEGF, FGF and TNF- α, although the mechanism of action is not yet fully understood (59, 69, 93). This drug was considered well-tolerated in canine patients at a daily dose of 10 mg/kg (94), although, it has typically been used at 2 to 4 mg/kg per day, in combination with cyclophosphamide and piroxicam (61, 69, 73, 81). Care must be taken with the timing of administration, as food intake seems to affect its bioavailability, delaying but increasing its absorption (95). Despite this, the accessibility of thalidomide on the global market has been limited due to the severe teratogenic effects reported in humans (96), which may compromise its routine use in veterinary metronomic protocols in some geographical territories. In turn, doxycycline is a tetracycline antibiotic with reported antiangiogenic and cytotoxic activity on tumour cells (97, 98). However, evidence on its specific therapeutic effect in canine and feline neoplastic conditions is still lacking, with recommended doses for anticancer purposes not yet being established.

### 3.3 Adverse events

Currently, the toxicity of antineoplastic therapies in dogs and cats is estimated based on adverse events (AEs), according to the criteria published by the Veterinary Cooperative Oncology Group (VCOG-CTCAE) (99). Each AE can be classified with a grade, according to its severity: grade 1 (mild), grade 2 (moderate), grade 3 (severe), grade 4 (life-threatening) and grade 5 (death). This classification system allows the clinician to define the recommended intervention according to the severity of each AE, as well as understand its impact on activities of daily living (ADL) and the consequences on the patient’s health status. Although this toxicity is typically low grade, several AEs have been reported in veterinary patients, with gastrointestinal signs, sterile haemorrhagic cystitis (SHC) and haematological toxicity being the most frequent (44, 62, 64, 68, 75, 86).

Regarding gastrointestinal toxicity, vomiting, diarrhoea, anorexia and nausea have been the main signs recorded in animals treated with metronomic cyclophosphamide (44, 56, 61–63, 65, 69–71, 73–77, 80–82), chlorambucil (83, 84), and lomustine (86). These signs tend to appear in the short term, typically within the first month of treatment, and are generally low grade (1 or 2) and self-limiting, requiring only supportive treatment (44, 62, 71, 73, 77, 79, 80, 83, 86). Moreover, NSAIDs have also been associated with gastrointestinal disturbances, especially piroxicam, which could potentially limit their long-term use in some cancer-bearing dogs and cats (92, 100). Still, it appears to be generally well-tolerated in feline patients even after one month and particularly if used as sole therapy (101).

In turn, sterile haemorrhagic cystitis has been described in dogs treated with oral metronomic cyclophosphamide, due to the formation of acrolein through liver metabolism, which accumulates and causes irritation in the bladder mucosa (55, 76, 90). This toxicity can affect up to 58% of canine patients (55, 59, 61, 62, 70, 73, 75–77, 81, 90) and must be prevented by administering it in the morning and encouraging water intake and frequent urination, in order to reduce urinary stasis. In line with this, the concomitant use of diuretics, such as furosemide, has also been advised (90, 102). Furthermore, if this urinary AE occurs, cyclophosphamide is generally replaced by chlorambucil (57, 70, 73, 74, 76, 80). The time required for its development differs depending on the dose of cyclophosphamide. Lower doses, such as 10 mg/m^2^, have been associated with a later onset of this AE, particularly when compared to doses of 15 to 25 mg/m^2^ (74, 75).

Haematological toxicity has been also associated with MC, as result of bone marrow suppression caused by the continued use of these drugs, and can be expressed as anaemia, thrombocytopenia and neutropenia of different grades (44, 56, 58, 62, 63, 65, 68, 86). These cytopenias are generally mild to moderate and transient, and may develop within the first few weeks or only after several months (44, 58, 86).

Finally, mild to severe renal toxicity has been reported in both dogs (65, 69, 81) and cats (56, 63, 82) treated with metronomic cyclophosphamide. This potential nephrotoxic effect may be worsened by the concomitant use of NSAIDs, such as piroxicam, requiring close monitoring, especially in older patients (100, 101).

Other undesired harmful effects may be described as the application of MC continues to increase in veterinary medicine, mainly with drugs whose toxicological profiles in companion animals have been less studied. For example, in human patients, continued administration of etoposide and thalidomide have been, respectively, associated with an increased risk of secondary leukaemia (103) and thromboembolic events (104), although a similar association has not yet been described in dogs and cats.

Despite all the potential AEs discussed above, it should be noted that MC has been associated with significantly fewer AEs than MTDC, as described by Marconato et al. (61) in a multi-institutional retrospective study (15.8% versus 43.5%, respectively).

The AEs reported in the various clinical trials published to date are described in detail in Table 2, along with the respective management.

**Table 2:** Adverse effects associated with metronomic chemotherapy in veterinary patients included in 36 clinical trials.

Reference/N° of animals treated with MC^†^	Main metronomic drug, dosage, schedule and duration	Percentage (%) of animals with adverse effects identified	Strategies adopted to manage adverse effects
Lana et al. (55) 9 dogs	CYC (12.5 to 25 mg/m^2^/day PO), with etoposide and piroxicam, for 6 months.	SHC in 22.2% (*n* = 2).	Drug discontinuation and treatment only with etoposide and piroxicam thereafter.
Elmslie et al. (77) 30 dogs	CYC (10 mg/m^2^/day or EOD PO), with piroxicam, on a long-term.	GI toxicity (grade 1 to 2) in 23.3% (*n* = 7); SHC (grade 2 to 4) in 10% (*n* = 3); and azotaemia (grade 2) in 6.7% (*n* = 2).	Drug frequency reduced from daily to EOD. Drug discontinuation in only 1 dog with grade 4 cystitis.
Tripp et al. (86) 52 dogs	Lomustine (2.84 mg/m^2^/day PO), associated with other therapies, for 98 days.	GI toxicity (grade 1 to 2) in 25%; ↑ ALT in 21.2%; thrombocytopenia (grade 1 to 4) in 23%; anaemia (grade 1 to 2) and azotaemia in 15.4% each; and neutropenia (grade 1) in 1.9%.	Dose reduction from daily to EOD (*n* = 2) or drug discontinuation (*n* = 22).
Burton et al. (43) 11 dogs	CYC (12.5 or 15 mg/m^2^/day PO), for 28 days.	No AEs were reported.	Not applicable.
Marchetti et al. (78) 15 dogs	CYC (25 mg/m^2^/day PO), with celecoxib, until recurrence/progression.	No AEs were reported.	Not applicable.
Leach et al. (83) 36 dogs	Chlorambucil (4 mg/m^2^/day PO) +/− NSAID, on a long-term.	GI toxicity (grade 1 to 2) in 11.1% (*n* = 4).	Supportive care for GI acute disorders.
Mitchell et al. (44) 13 dogs	CYC (15 mg/m^2^/day PO), with toceranib, for 4 to 6 weeks.	GI toxicity in 15.4%; neutropenia and thrombocytopenia (grade 1) in 7.7% each; and lethargy (grade 1 to 2) in 15.4%.	Reduction of toceranib dose and/or frequency when needed, but without adjustment of CYC.
Schrempp et al. (84) 31 dogs	Chlorambucil (4 mg/m^2^/day PO) +/− NSAID, on a long-term.	GI toxicity in 12.9%; lethargy (grade 1) in 6.5%; and haematological toxicity (grade 2 to 3) in 3.2% (*n* = 1).	Drug discontinuation in 1 dog with haematological toxicity.
Bracha et al. (71) 30 dogs (14 CM and 16 ACM)	CYC (10 to 12 mg/m^2^/day PO), with piroxicam and carboplatin (CM) or all plus DOX (ACM), on a long-term.	CM: GI (grade 1 to 3) and haematological (grade 1 to 4) toxicities; ACM: GI and haematological toxicities (grade 1 or 2).	Supportive care for GI toxicity and antibiotic therapy for haematological toxicity (grade 3 or more). Drug discontinuation (*n* = 6) and MTDC drug reduction (*n* = 6).
Leo et al. (56) 24 cats	CYC (14 mg/m^2^/day, EOD or twice weekly PO), with NSAID, toceranib +/− thalidomide, for at least 1 month.	GI toxicity (grades 1 to 2) in 16.7% (*n* = 4); haematological toxicity (grade 1 to 2) in 8.3% (*n* = 2); and renal toxicity in 4.2% (*n* = 1).	Supportive care +5-day drug holiday in one patient with vomiting; and metronidazole in one patient with diarrhoea.
Spugnini et al. (79) 22 dogs and 2 cats	CYC (12.5 mg/m^2^/day PO) in dogs and chlorambucil (4 mg/m^2^ EOD PO) in cats, with piroxicam, lansoprazole and a water alkaliser, until CR or absence of disease for 1 year.	Dogs: Mild GI toxicity in 50%, including diarrhoea (*n* = 1), vomiting (*n* = 2), and flatulence (*n* = 8). Cats: no AEs were reported.	Supportive care (*n* = 8) and lansoprazole dose reduction (*n* = 3).
London et al. (80) 81 dogs	CYC (10 mg/m^2^ EOD PO), with piroxicam (control_group_; *n* = 35) +/− toceranib (test_group_; *n* = 46), for 8 months or until signs of PD.	Test_group_: SHC in 10.9%; diarrhoea (grade 1 to 3) in 76.1%; vomiting (grade 1) in 28.3%; vomiting + diarrhoea (grade 4) in 2.2%; neutropenia (grade 1) in 30.4%; thrombocytopenia (grade 1) in 13%; ↑ ALT (grade 3) in 4.3%; weakness (grade 1 to 3) in 13%; and mild musculoskeletal pain/lameness in 17.4%/ Control_group_: SHC in 5.7%; vomiting + diarrhoea (grade 1) in 28.6 and 20%, respectively; thrombocytopenia (grade 1) in 22.9%; ↑ ALT + ↑ ALP (grade 4) in 2.9%; weakness (grade 1 to 2) in 8.6%; and mild musculoskeletal pain/lameness in 14.3%.	Supportive care in case of GI toxicity. CYC replaced by chlorambucil (*n* = 7) due to SHC. Toceranib dose reduction (*n* = 27) and temporary discontinuation (*n* = 10) due to toceranib-related AEs. Withdrawal in 9 dogs (8 test_group_ and 1 control_group_).
Wendelburg et al. (57) 26 dogs	CYC (9.2 to 16.0 mg/m^2^/day PO), with NSAID +/− etoposide (MC_group_) or DOX plus NSAID (MC + MTD_group_), on a long-term/until death.	Transient GI toxicosis in 3 of 7 dogs (42.9%) that received MTDC and MC concurrently.	Treatment with DOX and CYC were delayed when necessary (*n* = 1 and *n* = 2, respectively). In 1 case, CYC was replaced by chlorambucil (2 mg/m^2^/day PO).
Cancedda et al. (67) 20 dogs	CYC (7 mg/m^2^ EOD PO), with thalidomide, piroxicam and radiotherapy, on a long-term.	Only radiation-related AEs were reported.	Not applicable.
Rasmussen et al. (72) 13 (phase I) + 8 (phase II) dogs	CYC (10 to 15 mg/m^2^/day PO), combined or not with DOX, for a total median time of 84 days (phase I) and 42 days (phase II).	No AEs were reported.	Not applicable.
Finotello et al. (73) 10 dogs	CYC (7 to 15 mg/m^2^/day or EOD PO), with NSAIDs +/− thalidomide, on a long-term.	SHC (grade 2) in 20% (*n* = 2) and GI toxicity (grade 1) in 20%.	CYC replaced by chlorambucil (4 mg/m^2^/day or EOD PO) in both cases of SHC. Supportive care in GI cases.
Denies et al. (42) 30 dogs	CYC (12.5 mg/m^2^/day PO), temozolomide (6.6 mg/m^2^/day PO) or both, until death.	No AEs were reported.	Not applicable.
Cicchelero et al. (23) 6 dogs	CYC (12.5 mg/m^2^/day PO), with IL-12 EGT, until day 35.	Anorexia (grade 1) in 16.7%; tumour pain (grade 2) in 16.7%; and weight loss in 66.7%. Erythema/swelling with IL-12 EGT.	Coaxing/dietary change and tramadol (2 mg/kg PO) to manage loss of appetite and pain, respectively.
Matsuyama et al. (75) 50 dogs	CYC (25 mg/m^2^ EOD PO), for a median duration time of 90 days.	Anaemia (grade 1 to 3) in 38%; SHC in 32%; ↑ serum urea in 28%; ↑ ALT in 24%; ↑ creatinine in 14%; and GI toxicity (grade 2 to 3) in 14%.	Treatment discontinuation in 44% (*n* = 22).
Matsuyama et al. (74) 18 dogs	CYC (10, 15 or 25 mg/m^2^/day or EOD), with NSAID, for a total median time of 46 days.	SHC and ↑ serum urea in 16.7% each; and GI toxicity in 11.1%. Lethargy/collapse (*n* = 1) but probably related to progression.	CYC was replaced by chlorambucil (4 mg/m^2^/day PO) in two cases of SHC.
Duffy et al. (68) 29 dogs	Lomustine (2.84 mg/m^2^/day PO), combined or not with NSAID, until death or dose-limiting AEs.	Dose-limiting: thrombocytopenia (persistent grade 1); ↑ ALT (grade 3); and azotemia in 3.4% each. Non-dose-limiting: ↑ ALP (grade 1 to 3) in 17.2%; ↑ ALT (grade 1 to 2) in 6.9%; and transient grade 1 thrombocytopenia and diarrhoea in 3.4% each.	Discontinuation of lomustine or NSAID, depending on dose-limiting or non-dose-limiting toxicity, respectively.
Matsuyama et al. (76) 19 dogs	CYC (15 mg/m^2^/day PO), for a total median duration time of 94 days.	SHC (grade 2 to 3) in 57.9% (*n* = 11); GI toxicity (grade 1) in 10.5% (*n* = 2); and lethargy (grade 1) in 5.3% (*n* = 1).	CYC was replaced by chlorambucil (4 mg/m^2^/day PO) in 4 cases of SHC.
Bentley et al. (58) 8 dogs	Chlorambucil (4 mg/m^2^/day PO), prednisone and lomustine, for a median time of 258 days.	Chlorambucil-related AEs: Thrombocytopenia (grade 1 to 2) in 37.5%. Lomustine-related AEs: Neutropenia (grade 2) in 12.5%.	Chlorambucil dose reduction and/or discontinuation (*n* = 3). Lomustine dose reduction (*n* = 1).
Polton et al. (69) 25 dogs	CYC (10 mg/m^2^/day or EOD PO), with piroxicam and thalidomide, on a long-term.	GI toxicosis (grade 1 to 2) in 32% (*n* = 8); lethargy (grade 1) in 4% (*n* = 1); and renal toxicity (grade 3) in 4%.	Treatment discontinuation (*n* = 1) due to renal toxicity.
De Campos et al. (59) 9 dogs	CYC (15 mg/m^2^/day PO), with firocoxib, for 6 months.	SHC (44.4%; *n* = 4).	Drug interruption and treatment with prednisone (1 mg/kg PO for 10 days).
Alexander et al. (60) 22 dogs	CYC (12.5 mg/m^2^/day or 25 mg/m^2^ EOD PO), on a long-term.	Only MTDC-related AEs were reported.	Not applicable.
Marconato et al. (61) 38 dogs	CYC (10 to 15 mg/m^2^/day or EOD PO), with NSAID +/− thalidomide, for a median duration time of 35 days.	GI toxicity (grade 1 to 2) in 10.5% (*n* = 4); and SHC (grade 1) in 5.3% (*n* = 2).	No drug discontinuation or dose reduction was necessary.
Treggiari et al. (62) 43 dogs	CYC (10 to 15 mg/m^2^/day or EOD PO) or chlorambucil (4 mg/m^2^/day PO), with NSAID +/− thalidomide, on a long-term.	MC_group_ (*n* = 20): SHC (grade 1 to 2) in 20%; GI toxicity (grade 1 to 4) in 15%; and neutropenia (grade 2) in 5%. AMC_group_ (*n* = 23): GI toxicity (grade 1 to 3) in 47.8%; haematological toxicity (grade 1 to 3) in 34.8%; and SHC (grade 2 or 3) in 8.7%.	Supportive treatment, such as maropitant and mirtazapine to manage nausea/inappetence.
Marconato et al. (81) 6 dogs	CYC (10 mg/m^2^/day PO), with piroxicam and thalidomide, during a median time of 21 days.	GI toxicity (grade 1 to 2) in 50% (*n* = 3); SHC (grade 1) in 16.7% (*n* = 1); and renal toxicity in 16.7%.	Drug discontinuation and prescription of toceranib as a rescue option in one dog due to PD.
Petrucci et al. (63) 23 cats	CYC (15 mg/m^2^/day PO), with meloxicam, for 6 months.	Haematological toxicity (grade 1) in 13%; and GI (grade 1 to 2) and renal toxicity (grade 1 to 3) in 8.7% each.	No information available.
Petrucci et al. (82) 15 cats	CYC (15 mg/m^2^/day PO) or chlorambucil (0.4 to 0.6 mg/kg EOD PO), for 60 days (median).	Renal toxicity (grade 2) in 13.3% (*n* = 2); and GI toxicity (grade 1 to 2) in 13.3%; and anorexia (grade 1) in 6.7% (*n* = 1).	Dose reduction (10 mg/m^2^ EOD PO) in one cat due to GI toxicity.
Milevoj et al. (70) 12 dogs	CYC (15 to 25 mg/m^2^/day PO) +/− NSAID, for a median duration time of 156 days.	SHC (grade 1 to 3) in 33.3% (*n* = 4); GI toxicity (grade 1 to 2) in 25% (*n* = 3).	Withdrawal (*n* = 4) and replacement with chlorambucil (4 mg/m^2^/day PO; *n* = 3) due to SHC. Temporary discontinuation + supportive care, due GI toxicity.
Gedon et al. (85) 9 dogs	Chlorambucil (4 mg/m^2^/day PO), with meloxicam, for a median time of 297 days.	No AEs were reported.	Not applicable.
Machado et al. (64) 8 dogs	CYC (12.5 mg/m^2^/day PO), with carboplatin (300 mg/m^2^ IV), for 8 months.	Vomiting in 100%, considering at least 1 episode; and diarrhoea in 25%. Haematological toxicity was also detected.	Increased interval between carboplatin sessions (21 to 28 days) in 2 dogs, due to haematological toxicity.
Alonso-Miguel et al. (65) 8 dogs	CYC (12.5 mg/m^2^/day PO), with COX-2 inhibitor and toceranib phosphate, until death or dose-limiting AEs.	Haematological toxicity (grade 1 to 3) in 75%; GI toxicity (grade 1 to 3) in 62.5%; hypoalbuminemia (grade 1 to 2) in 25%; renal toxicity (grade 2 to 3) in 25%; and lethargy (grade 1) in 16.7%.	Temporary therapy discontinuation or complete withdrawal in 3 and 2 dogs, respectively.
Garcia et al. (66) 9 dogs	CYC (15 mg/m^2^/day or EOD PO), with tumour lysate vaccine, for 6 months.	No AEs were reported.	Not applicable.

AEs (adverse effects); ALP (alkaline phosphatase); ALT (alanine aminotransferase); COX (cyclooxygenase); CR (complete remission/response); CYC (cyclophosphamide); DOX (doxorubicin); EOD (every other day); GI (gastrointestinal); IL-12 EGT (intratumoral interleukin-12 electrogene therapy); IV (intravenous); MC (metronomic chemotherapy); MTDC (maximum-tolerated dose chemotherapy); NSAID (non-steroidal anti-inflammatory drug); PD (progressive disease); PO (per os/mouth); SHC (sterile haemorrhagic cystitis). Symbols: % (percentage); ↑ (elevation/ increase); ^†^number of animals assessed for toxicity.

### 3.4 Potential exposure hazards and safety measures

Unlike MTDC, which must be administered by a qualified veterinary professional in appropriate facilities with the necessary protective equipment, MC relies on oral administration of cytotoxic drugs to the animal in a home setting by the owner. Therefore, the person responsible for administering the drug is at greater risk of toxic exposure, which is particularly relevant with this type of chemotherapeutic protocols, as this procedure typically has to be performed daily for an extended period of time or even chronically (105). According to the International Agency for Research on Cancer (IARC), most cytostatics applied metronomically to canine and feline patients are considered carcinogenic to humans (group 1), such as cyclophosphamide, chlorambucil and etoposide, or at least probably carcinogenic (group 2A), such as lomustine (106). Therefore, to prevent health hazards, the owner must be adequately educated on the safety measures that must be followed when administering these medications, such as preserving the integrity of pills and capsules until adequate ingestion, using chemotherapy-rated gloves and washing hands afterwards (105). Although the potential risk of exposure through excretions, such as urine, faeces and vomit, has not been addressed in the literature in animals undergoing MC, care must be taken, and immediate cleaning using gloves is recommended (105, 107).

## 4 Clinical trials in dogs and cats

Since 2007, several clinical trials have been published on canine and feline patients treated with MC. According to the literature, this therapeutic approach has been applied to several tumour types, with splenic hemangiosarcoma (55, 57, 60–62, 73, 74) and appendicular osteosarcoma (68, 71, 76, 80) being the most common treated in dogs, followed by mammary carcinoma (59, 64, 65), soft tissue sarcoma (43, 67, 77), urinary tract tumours (84, 85), malignant oral tumours (66, 70), primary lung carcinoma (69), hepatocellular carcinoma (81) and cerebral glioma (58). In cats, there are much fewer studies available to date, focusing mainly on mammary tumours (63, 82). Additionally, some authors prescribed the same MC protocol to patients diagnosed with neoplasms of different histological types, evaluating their therapeutic response in a more heterogeneous group (23, 42, 44, 56, 72, 75, 78, 79, 83, 86).

Although they are not discussed in this article given the nature of the study (case report or case series) and/or the number of animals included (less than 5), there are descriptions of the use of MC in the treatment of cutaneous angiomatosis (108), intradural-extramedullary haemangioblastoma (109), malignant Leydig cell tumour (110), malignant mesenchymoma (111), maxillofacial osteossarcoma (112), omentum myxosarcoma (113), prostatic leiomyosarcoma (114) and tonsillar carcinomas (115) in dogs and abdominal (116) and urinary bladder (117) hemangiosarcomas in cats.

### 4.1 Canine splenic hemangiosarcoma

Total splenectomy is recommended in patients with splenic hemangiosarcoma, which has been associated with adjuvant chemotherapy protocols, particularly doxorubicin-based, in order to improve the prognosis of these patients (57, 118). More recently, several authors have suggested the addition of metronomic cyclophosphamide to the adjuvant treatment of these animals, but its potential therapeutic benefit is not yet completely clear, according to the literature. In fact, some authors have described a significant positive effect on prognosis (55, 73), whilst others have not (60–62, 74).

Two previous studies concluded that dogs with hemangiosarcoma treated with metronomic cyclophosphamide, either following conventional chemotherapy with doxorubicin (73) or as an alternative to it (55), lived significantly longer compared to those treated with adjuvant MTDC only. On the contrary, five others reported no significant improvement in outcome when MC was added to surgery (57), administered following surgery plus MTDC (60, 62, 74), or used as an alternative to adjuvant conventional chemotherapy (61, 62). Still, one of them suggested a therapeutic benefit at least in the short term (first 4 months) (57). There are also reports of the use of metronomic lomustine (86) and chlorambucil (62), but there is still no evidence to support its beneficial effect in these patients.

Therefore, as doxorubicin continues to be considered the only effective cytotoxic drug in the adjuvant treatment of this malignant neoplasm, MC should currently be reserved for cases in which the previous one is contraindicated (e.g., dogs with heart disease), or when a better quality of life with less therapy-related toxicity is prioritised at the potential expense of survival time (119).

### 4.2 Canine appendicular osteosarcoma

Amputation of the affected limb and subsequent carboplatin chemotherapy is the treatment of choice for appendicular osteosarcomas, but in order to improve its effectiveness, the adjuvant use of MC in these animals was investigated (68, 71, 76, 80). However, according to three studies, the addition of metronomic cyclophosphamide and an NSAID to conventional treatment did not offer any significant benefit in prolonging disease-free interval or survival time (71, 76, 80), not even when toceranib was also added (80). Metronomic lomustine has also been used in dogs with appendicular osteosarcoma, but despite being well tolerated (68, 86), it did not appear to significantly improve the survival of these patients, compared to other treatments such as radiotherapy (68). Thus, the data obtained so far do not support the use of MC in dogs diagnosed with appendicular osteosarcomas.

### 4.3 Canine mammary carcinoma

Mastectomy remains the treatment of choice for malignant mammary tumours in dogs, however adjuvant MTDC with single or multiple cytotoxic drugs, such as cyclophosphamide, 5-fluorouracil, mitoxantrone, carboplatin and gemcitabine, has been described for lesions at risk of local recurrence or metastasis (120). Given the complexity of these tumours, novel therapeutic approaches have been investigated, namely multi-targeted therapies, such as MC combined with NSAIDs.

In line with that, De Campos et al. and Machado et al. showed that metronomic cyclophosphamide following surgery and four (59) to six (64) cycles of intravenous carboplatin resulted in a better outcome than surgery alone (59) or surgery plus conventional chemotherapy only (59, 64). Interestingly, the only adjuvant drug that allowed a better prognosis in these patients when added instead of cyclophosphamide was thalidomide, according to one of these studies (59). Thus, according to these two studies, MC appears to be associated with a clinical benefit in the therapeutic management of canine mammary carcinomas.

In turn, inflammatory mammary carcinoma, the most aggressive form of mammary cancer in dogs, has been associated with a very poor prognosis despite the therapeutic approaches carried out, with no consensus regarding the benefit of chemotherapy (121). Even so, as these tumours were associated with a higher expression of COX-2, the use of its inhibitors, such as piroxicam, has been described (122, 123). In line with this, Alonso-Miguel et al. (65) recently evaluated the potential benefit of adding metronomic cyclophosphamide and toceranib phosphate to COX-2 inhibitor therapy alone, showing a significant increase in survival. However, the small number of dogs assessed and the retrospective nature of the study prevent further clinical conclusion. In fact, based on the current scientific evidence, an effective medical therapy has yet to be found and will probably be based on new therapeutic targets (121, 124).

### 4.4 Canine soft tissue sarcomas

Soft tissue sarcomas (STS) must be excised with wide margins in order to avoid local recurrence and potentially achieve therapeutic cure. In addition, adjunctive chemotherapy and/or radiotherapy protocols have been applied for incompletely resected and high-grade tumours, even though the level of scientific evidence is still considered low (125).

Nevertheless, metronomic cyclophosphamide has been described as effective in preventing the recurrence of these incompletely resected tumours, allowing longer disease-free times compared to surgery alone (77). A survival benefit was also reported by Cancedda et al. (67) in their retrospective study on dogs with macroscopic STS treated with hypofractionated radiotherapy followed or not by MC with oral cyclophosphamide, piroxicam and thalidomide. In this study, dogs that underwent adjuvant MC lived significantly longer (*p* = 0.023), although no significant difference in the progression-free interval was obtained. Metronomic use of chlorambucil was also evaluated in these patients. Leach et al. (83) reported that one dog with a STS of the flank was still in complete remission 35 weeks after starting this drug. According to these studies, MC seems to be a valid option in the adjuvant treatment of these patients.

### 4.5 Canine urinary tract tumours

The administration of chemotherapeutic agents, such as mitoxantrone, carboplatin or vinblastine, in combination with NSAIDs, has been considered the treatment of choice for urinary tract tumours in dogs due to the typically challenging location that often prevents surgery from being feasible (126).

Two studies described the metronomic use of chlorambucil in dogs diagnosed with malignant neoplasms affecting the bladder (84, 85) and/or urethra (85). According to Schrempp et al. (84), a chlorambucil-based metronomic protocol appears to be a well-tolerated and effective option for dogs with transitional cell carcinoma of the urinary bladder, particularly when other therapies have failed. More recently, Gedon et al. (85) showed that oral administration of chlorambucil combined with meloxicam in patients with urothelial carcinoma appears to be a good therapeutic option compared to NSAID treatment alone.

More studies are needed, but given these results, this protocol should be considered at least in three clinical scenarios: (1) when conventional chemotherapy has failed; (2) when an alternative chemotherapeutic approach with lower toxicity is intended; and (3) when NSAID therapy is elected, since the addition of metronomic chlorambucil appears to enhance its therapeutic effect.

### 4.6 Canine malignant oral tumours

Although surgery and/or radiotherapy are the preferred approaches to treat malignant oral tumours in dogs, oral metronomic cyclophosphamide could be beneficial as a palliative option when owners refuse them, as suggested by Milevoj et al. (70). In that study, half of the animals achieved partial response or stable disease after 1 month. However, it has several limitations, such as the small number of animals enrolled, the variability of histological types and the lack of a control group, which lower the level of evidence In contrast, Garcia et al. (66) found no clinical benefit in adding metronomic cyclophosphamide to an immunotherapy protocol in dogs with oral melanoma. Thus, randomised and controlled studies are needed to clarify the potential advantage of MC in oral malignant tumours in dogs.

### 4.7 Canine primary lung carcinoma

Surgical removal of primary pulmonary tumours is the therapeutic approach that allows a better prognosis, however given their location and size it may not be possible, requiring systemic treatment as alternative, such as MTDC with carboplatin, vinorelbine or gemcitabine (69, 127, 128). A MC protocol based on cyclophosphamide, piroxicam and thalidomide was also described by Polton et al. (69), which was associated with a significant therapeutic benefit in dogs diagnosed with advanced primary lung carcinoma. According to the multivariable survival analysis performed, patients who did not receive MC and underwent surgery, MTDC or no oncological treatment, had a 1.7 and 1.5 increased risk of tumour disease progression and death, respectively. Considering this outcome, the low toxicity reported and the improvement in quality of life described in most patients (91.3%), MC appears to be a good therapeutic alternative in unresectable and/or metastatic primary pulmonary tumours, although more studies are needed to strengthen this evidence.

### 4.8 Canine hepatocellular carcinoma

A single study was published on the therapeutic use of MC in hepatocellular carcinomas in dogs (81). The authors’ aim was to investigate an effective and well-tolerated chemotherapy alternative for this tumour type, as the prognosis is typically poor when complete surgical resection is not possible. However, animals treated with metronomic cyclophosphamide, piroxicam and thalidomide had a poor outcome. Therefore, a potentially effective systemic treatment (such as MC) for the management of canine hepatocellular carcinoma remains to be found.

### 4.9 Canine cerebral glioma

For the treatment of canine intracranial tumours, surgery and/or radiotherapy are the recommended options. By contrast, chemotherapy has a very limited therapeutic value given the heterogeneity of these tumours and the fact that the blood–brain barrier often compromises exposure to cytotoxic drugs in adequate doses (129). Even so, Bentley et al. (58) described the metronomic use of daily chlorambucil, associated with prednisone and monthly lomustine, after microsurgical resection of canine cerebral gliomas. This adjuvant approach was well tolerated and these patients had a better outcome compared to others previously treated with symptomatic approach and lomustine alone (130, 131). However, these promising results must be interpreted carefully given the small number of dogs enrolled in the study. Therefore, further studies are needed, not only to evaluate this protocol, but also to investigate the potential benefit of combining MC with other therapies typically recommended in these patients, such as adjuvant radiotherapy.

### 4.10 Feline mammary carcinoma

Feline mammary carcinomas are highly malignant, requiring an aggressive approach that typically involves radical mastectomy followed by doxorubicin (132, 133), carboplatin (134) or mitoxantrone (135). In order to find adjuvant alternatives associated with fewer AEs and that could avoid potentially stressful treatment sessions for cats, metronomic protocols have recently been suggested. However, according to these studies, female cats treated with metronomic cyclophosphamide (63, 82) or chlorambucil (82) did not live significantly longer than cats that underwent surgery alone (63), surgery plus doxorubicin-based MTDC (63, 82) or even surgery plus toceranib phosphate administration (82). Thus, to date, there is no scientific evidence to support the use of MC in this group of patients.

### 4.11 Canine and feline metastatic tumours

MC has also been applied in the treatment of canine metastatic tumours, whether with cyclophosphamide (78, 79), chlorambucil (83) or lomustine (86). Considering the typically guarded prognosis of these patients, promising results have been described with MC as some animals have achieved stable disease (78, 83, 86) or even partial (79, 86) and complete responses (78, 79). Feline advanced or metastatic tumours have also been treated with metronomic cyclophosphamide (56, 82) or chlorambucil (79), with some cats achieving stable disease as well (56). Therefore, according to these studies, MC should be considered in patients with metastatic neoplastic disease, not only as palliative therapy, but also as an adjuvant or even first-line approach, depending on the specific case.

## 5 Limitations of metronomic chemotherapy in clinical practice

The overall promising evidence discussed above, associated with greater affordability, lower risk of drug resistance and lower rate of adverse effects, explains the recent rise in popularity of MC in veterinary oncology.

However, there are still some factors at present that limit the use of MC in clinical practice and that should not be overlooked. Firstly, the lack of standardised dosing protocols and comprehensive clinical trials for specific tumour types pose challenges to its widespread clinical adoption. Secondly, whilst MC is generally associated with fewer and less severe side effects compared to traditional chemotherapy (61), long-term administration can still lead to cumulative toxicity in some patients, requiring careful monitoring and management (65, 75, 86). In addition, there may be a risk of developing chemoresistance over time, as resistance mechanisms to antiangiogenic drugs have been described in human medicine (136, 137). Lastly, this treatment approach will always depend on the adequate compliance of the owner who is responsible for administering the oral drug at home. In fact, the owner’s lack of compliance may be one of the main factors that continues to limit the application of MC in companion animals, namely due to the difficulty in administering one or more medications orally on a daily basis. This aspect may be particularly relevant in feline patients in whom medication at home has been considered more challenging and often associated with a negative experience for the owner and the cat (138). The existence of significantly fewer clinical trials on the use of MC in this species compared to dogs can be partially explained by this reported constraint.

In order to overcome these limitations, the commitment to further research will be crucial in optimising metronomic doses, assessing potential toxicity and exploring the full spectrum of therapeutic effects across a wider array of neoplastic diseases, ensuring that this treatment approach reaches its full potential.

## 6 Conclusions and forward directions

MC marks a revolutionary shift in the approach to cancer treatment, transitioning from conventional high-dose regimens to a strategy that prioritises a continuous and low-dose administration of chemotherapeutic agents. This method capitalises on the complex interactions within the TME, the process of angiogenesis, and the direct targeting of cancer cells, offering an alternative way of fighting malignancy. In line with that, its application has already shown therapeutic benefits in several neoplasms in dogs and cats, either as monotherapy or in combination with other treatment approaches, particularly in canine mammary carcinomas and canine soft tissue sarcomas.

Looking forward, the landscape of veterinary oncology is set to evolve significantly, with ongoing research aimed at refining MC protocols to identify the most effective dosing strategies. In addition, the integration of MC into new therapeutic protocols, including immunotherapy and targeted therapies, opens the possibility for personalised oncological care, contributing to better outcomes. This could pave the way for its more widespread clinical use in the management of various tumour types, including metastatic and otherwise incurable diseases, offering hope for extended survival and improved quality of life.

## Author contributions

GP: Conceptualization, Data curation, Formal analysis, Investigation, Writing – original draft. TM: Data curation, Formal analysis, Investigation, Validation, Writing – original draft. MD: Data curation, Investigation, Writing – original draft. FQ: Conceptualization, Funding acquisition, Project administration, Supervision, Writing – review & editing.
